# The association between socio-economic position and diet quality in rural and regional Australian adults

**DOI:** 10.1017/S0007114525103486

**Published:** 2025-06-28

**Authors:** Rebekah Pullen, Matthew J. Sharman, Ami Seivwright, Denis Visentin, Sebastian Kocar, Tracy Schumacher, Clare E. Collins, Elizabeth Lester, Katherine Kent

**Affiliations:** 1 School of Health Science, College of Health and Medicine, University of Tasmania, Launceston, TAS 7250, Australia; 2 Monash Climate Change Communication Research Hub, Monash University, Caulfield East, VIC 3145, Australia; 3 Centre for Longitudinal Studies, University College London, London, UK; 4 Department of Rural Health, University of Newcastle, Tamworth, NSW 2340, Australia; 5 University of Newcastle, School of Health Sciences, College of Health, Medicine and Well-being, Callaghan, NSW 2308, Australia; 6 Food and Nutrition Research Program, Hunter Medical Research Institute, New Lambton Heights, NSW 2305, Australia; 7 School of Medical, Indigenous and Health Sciences, Faculty of Science, Medicine and Health, University of Wollongong, Wollongong, NSW 2522, Australia

**Keywords:** Diet quality, Australian recommended food score, Healthy eating quiz, Index of social advantage and disadvantage, Australian adults

## Abstract

Diet quality has been linked to socio-economic status. However, evidence within rural and regional populations is lacking. This cross-sectional study examined the relationship between diet quality and socio-economic position in adults living in rural and regional areas of Australia. The Australian Recommended Food Score (ARFS; range 0–73) measured diet quality (total and subscale scores). Area-level socio-economic position was determined by postcode-linked socio-economic index for areas (SEIFA), Index of Relative Social Advantage and Disadvantage scores, stratified into quintiles. The mean total ARFS (34·7; sd = 9·1; *n* 836) was classified as ‘getting there’. Findings showed significantly lower mean total ARFS between SEIFA quintile 1 (1 = lowest; mean total ARFS = 30·4; sd = 10·2; categorised as ‘needs work’) compared with all other SEIFA quintiles (F (44 831) = 8·44, *P ≤* 0·001). Linear regression, adjusting for age, sex, income, education, employment status and household composition demonstrated significantly lower overall diet quality for SEIFA quintile 1 compared with SEIFA quintile 3 (B = –3·9; 95 % CI (–6·2, −1·5); *P* < 0·001) and lower subscale scores for vegetables (B = –1·6; 95 % CI (–2·7, −0·6); *P* = 0·003), fruit (B = –0·9; 95 % CI (–1·6, −0·1); *P* = 0·018) and grains (B = –0·6; 95 % CI (–1·3, −0·0); *P* = 0·050). After adjusting for individual confounders of diet quality, results indicate that lower area-level socio-economic position remained associated with poorer diet quality in this sample of rural and regional Australian adults. This suggests that broader social and environmental factors unique to these areas may impact diet quality and amplify individual barriers to achieving a healthy diet.

Socio-economic position refers to ‘social and economic factors that influence the position that individuals or groups hold within the structure of society’^(1)^ and as such serve to increase or decrease the likelihood of exposure to health-enhancing resources^(1)^. Krieger et al.^(2)^ proposed that socio-economic status, measured by characteristics such as occupation, wealth and consumption of goods and services, is an indicator of socio-economic position and can be measured on an individual, household or area level. At the area level, composite indices employ population-based census data to create a measure of socio-economic conditions of an area^(1)^. In Australia, area-level socio-economic position can be determined using *socio-economic indexes for areas* (*SEIFA*). The SEIFA Index of Relative Advantage and Disadvantage (IRSAD) describes access to material and social resources, such as income, education and housing, which impact people’s ability to participate in society^(3)^. Previous research has demonstrated an association between area-level socio-economic position, diet quality and prevalence of non-communicable diseases^(4)^. For example, in 2018, the disease burden attributable to dietary risk factors was reported to be twice as high among Australians of low socio-economic position compared with those of higher socio-economic position^(5)^. Further, in Australia, rural and regional locations are often lower socio-economic position^(6)^. As well as social and economic factors, vast geography and small populations mean that rural and remote areas experience greater barriers that prevent or limit access to adequate resources with which to improve social and economic outcomes^(7)^. Consequently, rural and regional populations experience lower life expectancy and a higher prevalence of non-communicable diseases, such as CVD, high blood pressure, obesity and type 2 diabetes compared with those in urban areas^(8)^.

Improving health outcomes for all Australians, regardless of socio-economic position or place of residence, could be supported by programs to facilitate adherence to the Australian Dietary Guidelines, which promote a pattern of eating within gender and age-based recommendations^(9)^. Globally and in Australia, higher diet quality has been associated with a lower risk of diet-related non-communicable diseases. For example, the Nurses’ Health Study in the USA found a 10 % increase in the Alternate Healthy Eating Index score in three prospective cohorts of females aged 30 – 55 years was associated with decreased risk of type 2 diabetes^(10)^. Similar results were reported for Australian women, with higher Alternate Healthy Eating Index scores associated with reduced odds of diabetes, hypertension, asthma and multimorbidity^(11)^. However, the relationship between living in rural and regional areas and diet quality varies across the globe^(12)^, and data on diet quality for rural and regional populations in the Australian setting are lacking. One Australian study comprising mostly regional and rural Australian adults^(13)^ found that diet quality was significantly lower for each additional diet-related non-communicable disease reported. Moreover, mortality from cardiovascular disease between rural and urban populations would be reduced by 38 % if regional and rural populations had similar diet quality as populations in metropolitan areas^(14)^.

Occupation, income and education are the three major individual-level socio-economic factors that impact diet quality^(15)^. However, even when controlling for these individual factors, differences in dietary patterns across geographic regions and socio-economic position persist^(16)^. Turrell and Kavanagh^(17)^ suggested that higher educational attainment may contribute to greater knowledge and understanding of nutrition and that occupation and income may impact food access and food choices. Specifically, people living in areas with limited access to social and economic resources, and therefore classified as being of lower socio-economic position, have been reported to consume more energy-dense, nutrient-poor foods due to their lower cost^(18)^. International evidence also demonstrates a positive association between lower socio-economic position and poor consumption of fruits and vegetables^(19)^. For rural and regional Australians, other environmental factors may include inadequate access and supply of healthy food^(20)^, and lengthy transit of perishable items over vast geographical distances often results in low quality or a lack of fresh foods^(21)^. Navigating the logistical challenges of food supply in rural and regional settings can limit the variety of types, sizes and brands of foods and also inflate costs^(21)^. The ‘local retail store’ is an important conduit to improving household nutrition in rural, regional and remote locations^(22)^. However, much of the research relating to the food environment, including health-enabling retail practices in these areas, has been conducted in Australian Aboriginal and Torres Strait Islander communities^(22)^. Therefore, the impact of the food environment in broader populations occupying rural and regional areas in Australia remains largely anecdotal^(22)^. Further, the measurement of nutrition and the food environment has relied heavily on the concept of a ‘healthy food basket’, which assess the accessibility of nutritious foods based on price and availability of predefined foods. However, this method is problematic in rural and regional areas due to exclusion of generic brands, a lack of stock for many of the predefined items and difficulty in assessing the quality of fresh food in these areas^(23)^.

To date, rural and regional nutrition research has not been prioritised in Australia^(24)^. Therefore, a significant knowledge gap exists regarding the impact of area-level socio-economic position on diet quality in rural and regional Australian populations. Focused studies of diet and nutrition outcomes in rural and regional areas have been identified as a priority for the next decade^(25)^. This study aimed to characterise diet quality in a sample of rural and regional Australian adults and examine the relationship between diet quality and socio-economic position. It was hypothesised that the diet quality of rural and regional Australian adults would be low and that individuals living in areas of low socio-economic position would have lower diet quality compared with those living areas of higher socio-economic position, even when controlling for individual-level socio-economic indicators.

## Methods

### Study design, setting and sample

This study was conducted in Tasmania, an island state of Australia that is considered regional, rural and remote under The Modified Monash Model, which categorises the remoteness of locations between 1 (major city) and 7 (very remote)^(26)^. In Tasmania, there are two main regional centres, Hobart and Launceston, which are Modified Monash Model category 2 (regional centres) in which 57 % of the population reside^(27)^, and the remaining regional cities and small towns are categories 3–7^(26)^. As part of The Tasmania Project (TTP), a survey of a convenience, non-probability sample of adult residents from all Tasmanian regions was conducted between 21 September and 9 October 2022 as part of TTP’s ‘Cost of Living’ survey^(28)^.

**Figure 1.**
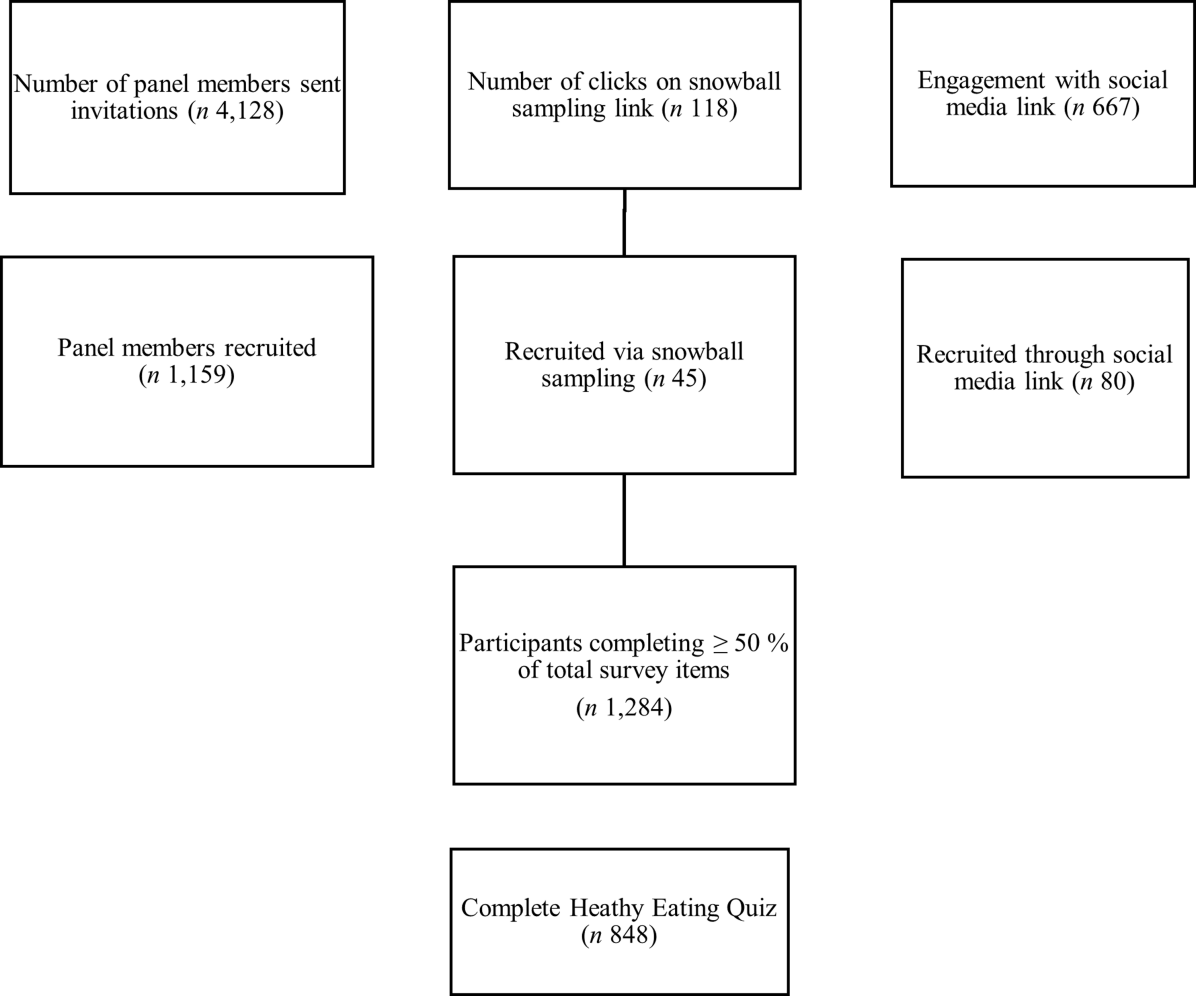
Flow chart of participant recruitment.

The current sample was recruited between April 2020 and September 2022 using several methods. The primary recruitment strategy included the use of a panel of preexisting volunteer participants who had previously participated in a TTP survey and provided an email address and consent to be contacted of future TTP surveys. Further, the project utilised email invitations to the University of Tasmania and other mailing lists, newspaper ads, online advertising via social media and snowball sampling. In total, 4128 participants from TTP’s panel were invited to complete the ‘Cost of Living’ survey, with 1159 completing at least 50 % of the questionnaire (Figure 1). Social media, including Twitter and Facebook, attracted 667 engagements, and from these, eighty individuals that were newly recruited completed the full survey (Figure 1). Snowball recruitment was undertaken via a survey link which participants could share with others who may have been interested. This link was utilised 118 times, with forty-five individuals completing at least 50 % of the questionnaire (Figure 1). Data were collected via online survey platform, Qualtrics (Qualtrics, Provo, UT). Completed survey responses were exported to IBM SPSS Statistics for Windows version 27.0 (IBM Corp.).

The study was conducted in accordance with the guidelines outlined in the Declaration of Helsinki and approved by the University of Tasmania Human Research Ethics Committee (Project ID 20587). STROBE guidelines were used for reporting in this manuscript. Eligibility criteria included being a resident of Tasmania and 18 years of age or over. These criteria were met by screening questions before participants were directed to a participant information sheet. Participant consent was obtained by responding to a question, ‘I have read and understood the Participant Information Sheet and I agree to take part in the project’.

### Diet quality

Dietary intake data were collected via the Healthy Eating Quiz, a 70-item online food frequency questionnaire^(29)^. The scoring of the Healthy Eating Quiz has been modelled from the Australian Recommended Food Score (ARFS), an extensively validated measure of diet quality^(30)^. The ARFS measures adherence to the Australian Dietary Guidelines, defined by variety and frequency, and provides an overall summary score (total ARFS). A higher ARFS represents usual intake of a greater variety of foods consumed from within the five food groups of the Australian Dietary Guidelines^(31)^. The ARFS has eight subscale scoring categories. The subscale categories (and scoring range) include vegetables (0–21 points), fruit (0–12 points), meat (0–7 points), plant-based protein (0–6 points), cereals/grains (0–13 points), dairy foods (0–11 points), water (0–1 point) and sauces (0–2 points). Subscale scores are summed to produce a total diet quality score (maximum of 73 points). This total diet quality score can then be categorised into the following groups: ‘needs work’ (< 33), ‘getting there’ (33–38), ‘excellent’ (39–46) and ‘outstanding’ (47+)^(31)^.

### Area-level socio-economic position

*Socio-economic indexes for areas* (*SEIFA*) and IRSAD were used to characterise area-level socio-economic position. Each participant’s postcode was matched to Tasmanian 2016 SEIFA decile rankings,^(3)^ which were collapsed into quintiles.

### Socio-demographic characteristics

Socio-demographic characteristics included sex, with binary response options of male and female. Other characteristics included age in years as a discrete variable categorised into six groups (18–24 years; 25–34 years; 35–44 years; 45–54 years; 55–64 years and 65 years and over). The highest level of educational attainment was collected and categorised into three levels (high school education or less; TAFE certificate or diploma (vocational education); or university level education). Postcode was categorised into Statistical Area level 4, the largest regional division within Tasmania^(32)^. Rurality was determined from postcode using the Modified Monash Model 2019 levels 1–7^(26)^. Aboriginal and/or Torres Strait Islander status was collected using binary Yes/No response options. Residency status was determined by categorising participants as Australian citizen, permanent resident, or temporary resident. Household composition (couple with/without non-dependent child(ren), person living alone, couple with dependent child(ren), single parent with dependent child(ren), multiple family household, non-related adults sharing and other) was recoded into a binary variable, living situation. The recoded variable consisted of those participants who cooked/ate alone (categories of ‘person living alone’ and ‘non-related adults sharing house/apartment/flat’) or cooked/ate with others (couple with/without non-dependent child(ren); couple with dependent child(ren); single parent with dependent child(ren); multiple family household and ‘other’). Weekly income data (AUD) were reported as negative income, which was defined as a person without an independent source of income, meaning their weekly income amounted to $0. Remaining categories were $1–$499, $500–$999, $1000–$1499, $1500–$1999, $2000–$2999 or $3000 or more. Employment status was collected and categorised as employed, worked full-time (including self-employed); employed, worked part-time (including self-employed); unemployed (including permanently unable to work); retired (including voluntarily inactive); performing home duties (caring for children and/or an ill or person with a disability); student; ‘other’ (please specify). Participants’ self-reported height (cm) and weight (kg) were used to calculate BMI (kg/m^2^), which was then categorised as underweight (< 18·5); normal weight (18·5–24·9); overweight (25·0–29·9); obesity I (30·0–34·9); obesity II (35·0–39·9) and obesity III (> 40)^(33)^.

### Statistical analyses

The final sample size comprised respondents for whom total ARFS and subscale category scores could be calculated and who had provided responses to socio-demographic questions, including valid postcode. Power analysis determined the minimum sample size required to test the study hypothesis. Results indicated the required sample size to achieve 80 % power for detecting a between-group difference of 3 points in total ARFS, at a significance criterion of α = 0·05, was *n* 113 IFA IRSAD quintile for the linear regression analyses performed

Statistical analysis was conducted using IBM SPSS Statistics for Windows, version 26.0 (IBM Corp.). Descriptive statistics were generated using cross-tabulation (frequencies and percentages) and were utilised to describe the distribution of socio-demographic characteristics, including SEIFA IRSAD quintiles in the sample. ARFS, socio-demographic variables were normally distributed and, therefore, were reported using mean and standard deviation. ARFS within levels of socio-demographic variables were compared using *χ* ^2^ tests. ANOVA with post hoc analysis using Bonferroni correction was used to conduct pairwise comparisons for mean total ARFS between SEIFA IRSAD quintiles.

Linear regression assessed the difference in ARFS mean total and subscale category scores according to SEIFA IRSAD quintiles, using quintile 3 as the reference category. The reference group (quintile 3) was selected to explore how disadvantaged or advantaged groups may compare to a median and to avoid comparing two extreme ends of this relative spectrum. Unadjusted and adjusted models were performed, with the adjusted model accounting for age, sex, education, income, employment status and living situation as covariates. These are all potential confounders of diet quality at the individual level^(4)^, thus adjusting for them facilitates the examination of the impact area-level socio-economic position as an independent mediator of diet quality. Confounders were established prior to statistical analysis. In both models, the ARFS subscale scores for water and sauces were excluded from the analysis as each subscale has only two individual scoring indicators.

Model assumptions generated through regression models were assessed. Durbin–Watson statistics were used to check for independence of observations. Variance inflation factors and Condition Index values were used to determine collinearity. All variables demonstrated a colinear relationship, with variance inflation factors statistic < 5. This was supported by condition index values below 15. Data for all variables included in the regression models demonstrated equal variance, homoscedastic characteristics and no significant outliers. This was established using partial regression plots, distributions of standardised residuals, Histograms and P-P plots. Regression models were found to have satisfied all model assumptions.

## Results

### Socio-demographic characteristics by SEIFA IRSAD quintiles

In the TTP Cost of Living survey, a total of 1284 participants were recruited. Of these participants, 66 % (*n* 848) provided data on diet quality (Table 1). Respondents were predominantly female (73·1 %), aged ≥ 65 years (31·8 %), with a university-level education (66·3 %), and incomes ranging between (AUD) $500 and $1000 per week (24·9 %). Most participants lived in inner regional areas, (62·8 %), followed by rural/remote (32·0 %) and outer regional areas (5·2 %). Most participants reported living in couple households (either with or without non-dependent children). The highest proportion of participants had a BMI classified as normal weight (35·6 %) followed by overweight (34·0 %) (Table 1). In comparison to the total sample recruited (*n* 1284), the sample of respondents who provided data to calculate an ARFS (*n* 848) were slightly older (55·1 years *v*. 54·0 years) and had a slightly higher proportion of female respondents (73·2 % *v*. 69·9 %).

**Table 1.** Socio-demographic characteristics for total sample and by Socio-economic Indexes for Areas, Index of Relative Social Advantage and Disadvantage quintile categories (*n* = 848)

		SEIFA IRSAD		SEIFA IRSAD		SEIFA IRSAD		SEIFA IRSAD		SEIFA IRSAD		SEIFA IRSAD		
		Total sample		Quintile 1		Quintile 2		Quintile 3		Quintile 4		Quintile 5		
		*n*	%	*n*	%	*n*	%	*n*	%	*n*	%	*n*	%	*P*-value (χ^2^)
Total		836		114	13·6 %	140	16·7 %	115	13·8 %	166	19·9 %	301	36·0 %	
Sex *n* = 826	Male	222	26·9 %	29	13·1 %	38	17·1 %	27	12·2 %	47	21·2 %	81	36·5 %	0·93
	Female	604	73·1 %	84	13·9 %	101	16·7 %	86	14·2 %	118	19·5 %	215	35·6 %	
Age category (years) *n* = 836	18–24	36	4·3 %	8	22·2 %	2	5·6 %	2	5·6 %	4	11·1 %	20	55·6 %	< 0·001
	25–34	66	7·9 %	14	21·2 %	9	13·6 %	10	15·2 %	6	9·1 %	27	40·9 %	
	35–44	110	13·2 %	25	22·7 %	12	10·9 %	12	10·9 %	30	27·3 %	31	28·2 %	
	45–54	150	17·9 %	15	10·0 %	28	18·7 %	17	11·3 %	32	21·3 %	58	38·7 %	
	55–64	208	24·9 %	28	13·5 %	48	23·1 %	32	15·4 %	38	18·3 %	62	29·8 %	
	≥ 65	266	31·8 %	24	9·0 %	41	15·4 %	42	15·8 %	56	21·1 %	103	38·7 %	
Education *n* = 836	High School	107	12·8 %	26	24·3 %	15	14·0 %	15	14·0 %	19	17·8 %	32	29·9 %	< 0·001
	TAFE/Diploma	175	20·9 %	34	19·4 %	40	22·9 %	28	16·0 %	28	16·0 %	45	25·7 %	
	University	554	66·3 %	54	9·7 %	85	15·3 %	72	13·0 %	119	21·5 %	224	40·4 %	
Rurality *n* = 806	Inner regional	506	62·8 %	64	12·6 %	90	17·8 %	63	12·5 %	97	19·2 %	192	37·9 %	0·23
	Outer regional	42	5·2 %	3	7·1 %	5	11·9 %	7	16·7 %	8	19·0 %	19	45·2 %	
	Rural/Remote	258	32·0 %	45	17·4 %	41	15·9 %	40	15·5 %	53	20·5 %	79	30·6 %	
Aboriginality *n* = 831	Not Aboriginal or Torres Strait Islander	801	96·4 %	108	13·5 %	132	16·5 %	114	14·2 %	160	20·0 %	287	35·8 %	0·33
	Aboriginal and/or Torres Strait Islander	30	3·6 %	5	16·7 %	7	23·3 %	1	3·3 %	4	13·3 %	13	43·3 %	
Residency status *n* = 836	Australian citizen	789	94·4 %	97	12·3 %	136	17·2 %	110	13·9 %	157	19·9 %	289	36·6 %	< 0·001
	Permanent resident	29	3·5 %	9	31·0 %	3	10·3 %	5	17·2 %	4	13·8 %	8	27·6 %	
	Temporary resident	18	2·2 %	8	44·4 %	1	5·6 %	0	0·0 %	5	27·8 %	4	22·2 %	
Household composition *n* = 836	Couple with/without non-dependent child(ren)	392	46·9 %	50	12·8 %	73	18·6 %	57	14·5 %	81	20·7 %	131	33·4 %	0·16
	Person living alone	190	22·7 %	31	16·3 %	26	13·7 %	32	16·8 %	28	14·7 %	73	38·4 %	
	Couple with dependent child(ren)	135	16·1 %	11	8·1 %	24	17·8 %	15	11·1 %	33	24·4 %	52	38·5 %	
	Single parent with dependent child(ren)	24	2·9 %	4	16·7 %	7	29·2 %	2	8·3 %	6	25·0 %	5	20·8 %	
	Multiple family household	40	4·8 %	8	20·0 %	5	12·5 %	4	10·0 %	9	22·5 %	14	35·0 %	
	Non-related adults sharing	28	3·3 %	3	10·7 %	3	10·7 %	2	7·1 %	4	14·3 %	16	57·1 %	
	Other	27	3·2 %	7	25·9 %	2	7·4 %	3	11·1 %	5	18·5 %	10	37·0 %	
Income p/w (AUD) *n* = 816	Negative income	10	1·2 %	0	0·0 %	2	20·0 %	3	30·0 %	2	20·0 %	3	30·0 %	0·02
	$0	26	3·2 %	2	7·7 %	4	15·4 %	7	26·9 %	2	7·7 %	11	42·3 %	
	$1–$499	164	20·1 %	39	23·8 %	27	16·5 %	24	14·6 %	24	14·6 %	50	30·5 %	
	$500–$999	203	24·9 %	28	13·8 %	32	15·8 %	24	11·8 %	48	23·6 %	71	35·0 %	
	$1000–$1499	165	20·2 %	21	12·7 %	26	15·8 %	24	14·5 %	37	22·4 %	57	34·5 %	
	$1500–$1999	121	14·8 %	12	9·9 %	19	15·7 %	21	17·4 %	26	21·5 %	43	35·5 %	
	$2000–$2999	96	11·8 %	9	9·4 %	15	15·6 %	9	9·4 %	16	16·7 %	47	49·0 %	
	$3000 or more	31	3·8 %	2	6·5 %	8	25·8 %	1	3·2 %	9	29·0 %	11	35·5 %	
Employment status *n* = 827	Full-time (including self-employed)	232	28·1 %	30	12·9 %	43	18·5 %	26	11·2 %	53	22·8 %	80	34·5 %	0·002
	Part-time (including self-employed)	178	21·5 %	22	12·4 %	31	17·4 %	25	14·0 %	36	20·2 %	64	36·0 %	
	Unemployed (and/or permanently unable to work)	34	4·1 %	7	20·6 %	9	26·5 %	10	29·4 %	3	8·8 %	5	14·7 %	
	Retired (including voluntarily inactive)	250	30·2 %	22	8·8 %	40	16·0 %	43	17·2 %	47	18·8 %	98	39·2 %	
	Performing home duties (caring for children and/or an ill person or person with a disability)	20	2·4 %	7	35·0 %	3	15·0 %	1	5·0 %	3	15·0 %	6	30·0 %	
	Student	57	6·9 %	12	21·1 %	5	8·8 %	7	12·3 %	9	15·8 %	24	42·1 %	
	Other	56	6·8 %	13	23·2 %	8	14·3 %	2	3·6 %	13	23·2 %	20	35·7 %	
BMI category *n* = 807	Underweight	10	1·2 %	1	10·0 %	2	20·0 %	1	10·0 %	3	30·0 %	3	30·0 %	< 0·001
	Normal weight	287	35·6 %	35	12·2 %	32	11·1 %	38	13·2 %	46	16·0 %	136	47·4 %	
	Overweight	274	34·0 %	31	11·3 %	49	17·9 %	38	13·9 %	65	23·7 %	91	33·2 %	
	Obesity I	133	16·5 %	23	17·3 %	25	18·8 %	18	13·5 %	28	21·1 %	39	29·3 %	
	Obesity II	55	6·8 %	11	20·0 %	17	30·9 %	9	16·4 %	8	14·5 %	10	18·2 %	
	Obesity III	48	5·9 %	8	16·7 %	12	25·0 %	3	6·3 %	12	25·0 %	13	27·1 %	

SEIFA, socio-economic index for areas; IRSAD, index of relative social advantage and disadvantage, a measure that ranks geographical locations by assessing the presence or absence of social advantage and disadvantage; Quintile 1 most disadvantage and least advantaged, quintile 5 most advantage least disadvantaged. *P* value derived from a chi-square test.

A total of 836 participants provided geographic information that allowed categorisation into SEIFA IRSAD quintiles, with quintile subgroups ranging from *n* 117 to *n* 220 per group. Of the total sample, 30·3 % of participants reported residing in SEIFA IRSAD quintiles 1 and 2 (highest disadvantage, lowest advantage) regions. Quintile 5 (highest advantage, lowest disadvantage) reported the highest proportion of participants (36·0 %; (Table 1). The proportions of participants across age categories varied for SEIFA IRSAD quintiles (Table 1)

SEIFA IRSAD scores are calculated from combined indicators of disadvantage and advantage such as household income, employment status and educational achievement. A geographical area with all indicators of advantage/disadvantage equal to the national average is allocated a SEIFA score of 1000. Regions with high SEIFA scores (above 1000) report indicators of advantage that are higher; or indicators of disadvantage that are lower than the national average. Conversely, regions with low SEIFA scores report indicators of disadvantage that are greater than the national average; or indicators of advantage that are below the national average. Given this, the significant differences between SEIFA quintiles for variables education, income and employment status seen in the present study were expected and reflect the construction of the SEIFA IRSAD index^(34)^ (Table 1). There were no significant differences in the proportions of participants across SEIFA IRSAD quintiles for the socio-demographic characteristics of household composition, rurality and aboriginality.

### Diet quality scores by SEIFA IRSAD quintiles

The mean total ARFS for the sample was 34·7 (sd = 9·1) (score range 0–73), which is classified as ‘getting there’ (Table 2). Participants in SEIFA IRSAD quintile 1 reported the lowest mean total ARFS (mean = 30·4, sd = 10·2), classified as ‘needs work’. Participants in SEIFA IRSAD quintile 5 reported the highest mean total ARFS (mean 35·6, sd = 8·6), which is defined as ‘getting there’ (Table 2). The results of one-way ANOVA significant differences in mean total ARFS between SEIFA IRSAD quintiles. Based on the Levene’s test, the outcome variable was normally distributed and equal variances are assumed ((F (4831) = 1·861, *P* = 0·115)). There was a significant difference between quintile 1 and each of the other SEIFA IRSAD quintiles ((F (44 831) = 8·44, *P* =< 0·001) and an effect size of η^2^ = 0·4. Results indicate that quintile 1 participants had significantly lower mean total ARFS in all pairwise comparisons, with differences in means ranging from −4·1 to −5·7 ARFS points (online Supplementary Table 1). Further analysis using one-way ANOVA demonstrated that there were no significant differences in mean total ARFS between quintiles 2–5.

**Table 2.** Australian Recommended Food Score (ARFS) means and standard deviation for total and sub-scale scores by Socio-economic Indexes for Areas, Index of Relative Social Advantage and Disadvantage quintiles

					SEIFA IRSAD Quintile									
ARFS and sub-scales	Reference range	*n*	Total sample		1		2		3		4		5	
			Mean	sd	Mean	sd	Mean	sd	Mean	sd	Mean	sd	Mean	sd
Total score	0–73	836	34·7	9·1	30·4	10·2	34·5	8·9	35·3	8·9	36·1	8·8	35·6	8·6
Vegetables	0–21	836	13·5	4·2	11·7	4·5	13·7	4·0	13·9	4·1	13·8	4·2	13·8	4·0
Fruit	0–12	835	5·1	2·7	4·1	2·8	5·0	2·9	5·1	2·6	5·4	2·5	5·3	2·6
Meat	0–7	737	3·0	1·5	2·6	1·5	3·3	1·4	2·8	1·6	3·3	1·5	2·9	1·5
Vegetarian sources of protein	0–6	737	2·7	1·4	2·3	1·3	2·6	1·3	2·9	1·4	2·9	1·4	2·8	1·4
Vegetarian proteins (Vegetarians only)	0–13	99	8·2	2·8	8·1	3·6	8·0	2·5	9·2	2·5	7·6	2·8	8·1	2·8
Grains	0–13	836	5·5	2·4	5·1	2·5	5·2	2·6	5·7	2·2	5·7	2·5	5·6	2·2
Dairy	0–11	836	3·1	1·8	2·9	1·9	3·0	1·9	2·8	1·8	3·3	1·9	3·2	1·8
Water	0–1	836	0·7	0·5	0·7	0·5	0·5	0·5	0·7	0·5	0·8	0·4	0·7	0·5
Sauces	0–2	836	0·8	0·7	0·7	0·7	1·0	0·8	0·8	0·8	0·9	0·8	0·7	0·8

SEIFA, socio-economic index for areas; IRSAD, index of relative social advantage and disadvantage, a measure that ranks geographical locations by assessing the presence or absence of social advantage and disadvantage; ARFS, Australian Recommended Food Score. Further information on scoring of ARFS can be found^(31)^; Quintile 1 most disadvantage and least advantaged, quintile 5 most advantage and least disadvantaged.

Participants within SEIFA IRSAD quintile 1 reported the lowest mean ARFS for subscale categories of vegetables (11·7/21 possible points), fruit (4·1/12 possible points), meat (2·6/7 possible points), vegetarian sources of protein (2·3/6 possible points) and grains (5·1/13 possible points) (Table 2). For the remaining ARFS subscale categories of dairy and vegetarian proteins, quintile 3 reported the lowest mean ARFS score for the subscale category dairy (2·8/11 possible points), and quintile 4 scored lowest for ARFS vegetarian proteins (vegetarians only) (7·6/13 possible points).

The unadjusted regression model (Table 3) demonstrates that SEIFA IRSAD quintile 1 had a significantly lower total ARFS (–4·9 points) when compared with the reference category SEIFA IRSAD quintile 3. There was, however, no significant difference between SEIFA IRSAD quintiles 2, 4 and 5 and the reference category (quintile 3) for total ARFS (Table 3). In the regression model adjusted for age, sex, education, income and living situation, the difference between total ARFS for SEIFA IRSAD quintile 1 was significantly lower (3·9 points) compared with the reference category (Table 3).

**Table 3.** Regression results for diet quality score by Socio-economic Indexes for Areas, Index of Relative Social Advantage and Disadvantage quintiles, unadjusted and adjusted for age, sex, education, income, employment status, BMI and living situation

Unadjusted						Adjusted for age, sex, education, income, employment status and living situation			
Diet quality score	SEIFA	B	se	95 % CI	*P* value	B	se	95 % CI	*P* value
	1	–4·9	1·2	–7·2, −2·5	< 0·001	–3·9	1·2	–6·2, −1·5	0·001
	2	–0·8	1·1	–3·0, 1·5	0·506	–1·4	1·2	–3·6, 0·9	0·241
Total	Quintile 3 Reference					Quintile 3 Reference			
	4	0·9	1·1	–1·3, 3·0	0·429	0	1·1	–2·2, 2·2	0·998
	5	0·3	1	–1·6, 2·2	0·751	–0·3	1	–2·3, 1·7	0·757
	1	–2·2	0·5	–3·3, 1·1	0·001	–1·6	0·6	–2·7, –0·6	0·003
	2	–0·2	0·5	–1·2, 0·8	0·669	–0·5	0·5	–1·5, 0·6	0·394
Vegetables	Quintile 3 Reference					Quintile 3 Reference			
	4	–0·2	0·5	–1·1, 0·8	0·736	–0·4	0·5	–1·4, 0·6	0·417
	5	–0·1	0·5	–1·0, 0·8	0·811	–0·3	0·5	–1·2, 0·6	0·557
	1	–1·0	0·3	–1·7, –0·4	0·003	–0·9	0·4	–1·6, –0·1	0·018
	2	–0·1	0·3	–0·8, 0·5	0·722	–0·5	0·3	–1·1, 0·2	0·194
Fruit	Quintile 3 Reference					Quintile 3 Reference			
	4	0·3	0·3	–0·3, 1·0	0·305	0	0·3	–0·7, 0·6	0·930
	5	0·2	0·3	–0·4, 0·8	0·472	0	0·3	–0·6, 0·6	0·926
	1	–0·2	0·2	–0·6, 0·2	0·383	0·1	0·2	–0·3, 0·5	0·655
	2	0·5	0·2	0·1, 0·9	0·012	0·5	0·2	0·1, 0·9	0·011
Meat	Quintile 3 Reference					Quintile 3 Reference			
	4	0·5	0·2	0·1, 0·9	0·009	0·4	0·2	0·1, 0·8	0·027
	5	0·1	0·2	–0·3, 0·4	0·584	0·1	0·2	–0·3, 0·5	0·595
	1	–0·5	0·2	–0·9, −0·1	0·006	–0·4	0·2	–0·8, 0·0	0·065
	2	–0·2	0·2	–0·6, 0·1	0·189	–0·3	0·2	–0·6, 0·1	0·193
Meat Alternatives (not vegetarian)	Quintile 3 Reference					Quintile 3 Reference			
	4	0	0·2	–0·3, 0·4	0·964	–0·1	0·2	–0·5, 0·3	0·607
	5	0	0·2	–0·4, 0·3	0·850	–0·1	0·2	–0·4, 0·3	0·663
	1	–1·1	1·2	–3·4, 1·3	0·362	–1·7	1·4	–4·5, 1·2	0·246
	2	–1·2	1·1	–3·5, 1·0	0·290	–0·4	1·5	–3·5, 2·7	0·796
Meat Alternatives (vegetarian)	Quintile 3 Reference					Quintile 3 Reference			
	4	–1·6	1·0	–3·7, 0·4	0·120	–1·1	1·3	–3·7, 1·5	0·412
	5	–1·1	0·8	–2·6, 0·4	0·144	–1·6	1	–3·6, 0·3	0·103
	1	–0·6	0·3	–1·2, 0·0	0·055	–0·6	0·3	–1·3, 0·0	0·050
	2	–0·5	0·3	–1·1, 0·1	0·102	–0·6	0·3	–1·2, 0·0	0·071
Grains	Quintile 3 Reference					Quintile 3 Reference			
	4	0	0·3	–0·5, 0·6	0·90	0	0·3	–0·6, 0·5	0·873
	5	–0·1	0·3	–0·6, 0·4	0·80	–0·3	0·3	–0·8, 0·3	0·318
	1	0·1	0·2	–0·4, 0·6	0·671	0·1	0·3	–0·4, 0·6	0·622
	2	0·2	0·2	–0·2, 0·7	0·295	0·2	0·3	–0·2, 0·7	0·320
Dairy	Quintile 3 Reference					Quintile 3 Reference			
	4	0·5	0·2	0·0, 0·9	0·038	0·4	0·2	–0·1, 0·8	0·123
	5	0·4	0·2	0·0, 0·8	0·034	0·4	0·2	–0·1, 0·8	0·100

SEIFA, socio-economic index for areas; IRSAD, index of relative social advantage and disadvantage, a measure that ranks geographical locations by assessing the presence or absence of social advantage and disadvantage; se, standard error; 95 % CI, 95 % confidence interval.

In the unadjusted model, SEIFA IRSAD quintile 1 reported significantly lower ARFS scores than the reference category for ARFS subscale for vegetables, fruit and meat alternatives (non-vegetarian). In the adjusted model (age, sex, education, income, employment status and living situation), the subscale score for vegetables (–1·6 points), fruit (–0·9 points) and grains (–0·6 points) were significantly lower in quintile 1 compared with the reference category (SEIFA IRSAD quintile 3) (Table 3). However, SEIFA IRSAD quintile 1 ARFS score for meat alternatives (non-vegetarian) did not remain significantly different in the adjusted regression model (Table 3). Participants in quintiles 2 and 4 reported significantly higher ARFS for the meat subscale category, which remained significant in the adjusted model. Further, the unadjusted regression demonstrated that quintiles 4 and 5 had significantly higher ARFS than the reference category for the subscale category of dairy. However, this result did not persist in the adjusted model.

## Discussion

To our knowledge, this study is the first to examine the association between area-level socio-economic position and a validated diet quality measure in a sample of rural and regional Australian adults. Our results indicate that the total diet quality reported is low, similar to other studies reporting diet quality in broader Australian samples^(31)^. Our findings also provide evidence that rural and regional Australians living in areas characterised by low socio-economic position have significantly lower overall diet quality, driven by low variety in fruit, vegetables and grains consumption compared with those living in all other quintiles, even when controlling for individual-level factors. This study fills a critical gap in knowledge of diet quality in regional and rural populations^(35)^.

The overall diet quality score of our sample (34·7 points) is similar to other studies that have reported mean total ARFS ranging from 33·9 points to 34·5 points^(13,31,36)^. Our results showed that all food groups, except for the vegetable category demonstrated poor ARFS subscale scores. This is in line with previous research in which poor diet quality scores are driven by non-adherence to most, if not all of the five food groups recommended in the Australian Dietary Guidelines^(37)^. These findings highlight the urgent need for targeted interventions and policy initiatives to address the specific challenges faced by rural and regional communities in being able to sustain dietary patterns that align with recommendations in the Australian Dietary Guidelines.

Our study identified that those living in the areas of highest relative disadvantage had significantly lower diet quality compared with all other quintiles (quintiles 2–5), who demonstrated similar diet quality scores. The similarity in overall diet quality scores for 80 % of SEIFA quintiles (quintiles 2–5) in this sample may suggest little difference in the relative access to social and economic resources in these regions, including the accessibility, availability and affordability of healthy foods. Conversely, the magnitude of difference in total diet quality for the remaining 20 % (quintile 1) demonstrates that disparities in access to social and economic resources appear to exist in areas characterised by the lowest socio-economic position, even after adjusting for individual levels factors like income and education, suggesting that area-level factors impact diet quality. The significant four-point difference in total diet quality score for people in areas of the highest disadvantage found in the current study is similar to two other Australian studies that reported significant differences of 3·0^(38)^ and 3·6^(39)^ Dietary Guideline Index points (range; 0–130) between the lowest and highest categories of SEIFA. Additionally, Williams et al.^(31)^ found a comparable decrease of 3·9 ARFS points for those in the lowest SEIFA decile *v*. the highest. However, their sample did not differentiate between metropolitan and regional and rural populations. Notably, total ARFS scores in the Williams et al.^(31)^ study for the lowest decile of SEIFA were 2·9 points higher than that of the lowest SEIFA quintile observed in the current rural and regional sample. Further, our adjusted model demonstrated that these differences in diet quality by area level disadvantage persisted even when controlling for individual socio-economic variables. This suggests that diet quality of people living in rural and regional areas may be poorer due to other area-level social, environmental and geographical factors like poorer access to education and limited exposure to health promotion initiatives and broader factors that negatively impact the community food environment such as fewer fresh food outlets and higher food prices^(40)^.

The finding of low variety in fruit, vegetables and grains subscale categories among people of low socio-economic position aligns broadly with evidence showing higher intakes of whole grains and fresh fruits and vegetables in those defined as having a higher socio-economic position^(4)^. However, a recent systematic review of Australian research shows inconsistencies in the food groups that are influenced by area-level socio-economic position^(41)^. Livingstone et al.^(39)^ found no association between area-level socio-economic position and fruit and vegetable variety, whereas Grech et al.^(42)^ found associations for fruit but not for vegetables. These findings further underscore the complexity of relationships between socio-economic factors and dietary patterns in different communities across Australia, and interventions aimed at improving diet quality in disadvantaged regions should ensure they are targeted to the needs and characteristics of each specific community.

The traditional dietary patterns in rural and regional Australians are characterised by a lack of variety in vegetable intake^(43)^, which is also evident in our study. Despite recognising the importance of fruits and vegetables in a healthy diet, those facing high relative disadvantage may prioritise other competing social and economic interests, such as shelf life and accessibility^(44)^. Geographic barriers to accessibility may also explain the effect of area-level disadvantage on diet quality over and above individual socio-economic position. Further, the food preferences of populations of low socio-economic position are determined at times by necessity and not preference, influenced greatly by foods considered most filling and cost efficient^(45)^. The limited variety of both fruits and vegetables in the current study sample may also relate to rapid inflation rates experienced in Australia in 2023, following disruptions to global food supplies related to the COVID-19 pandemic, natural disasters and global conflicts. Lewis et al.^(46)^, reported a 17·9 % increase in the cost of a healthy diet during this period. Specifically, fruits and vegetables increased in cost by 12·8 %^(46)^, which disproportionately affected people living in rural and regional areas of Australia^(47)^. With economists forecasting continued cost of living increases, further research into the impact of inflation on the ability of regional and rural Australians to maintain a healthy diet will be imperative.

In line with other research, the current findings also point to significantly lower variety in grain-based foods in people living in areas of high relative disadvantage^(31,42)^. For example, other research suggests that populations of low socio-economic position have the increased intake of refined grains^(4)^. Livingstone et al.^(39)^ also found this, reporting differences in wholegrain intake between lowest and highest SEIFA deciles. However, unlike our results, their study found no difference in overall intake of cereals. In Australia, recent research reports there is low overall grain consumption^(48)^. Our findings extend this, by showing that the rural and regional adults in the current sample regularly selected less than 50 % of the types of grain-based foods recommended by the Australian Dietary Guidelines. Notably, research indicates that adding two to three daily servings (45 g) of whole grains per day may be a favourable public health goal, with suggested benefits of reduce the risk of Type 2 diabetes, coronary heart disease, CVD and stroke^(49)^. Such an outcomes highlight the need to address dietary disparities, particularly in rural and regional populations, to mitigate chronic disease risk. To achieve such outcomes, targeted promotion of national dietary recommendations is needed to create awareness of strategies that could improve the health and well-being of populations in greatest need. Brimblecombe et al.^(50)^ demonstrated a 12·7 % increase in the sales of fruit and vegetables due to price discounts and a further 7·5 % increase when delivered alongside consumer educational strategies.

In contrast to findings of no differences in variety of meat, meat alternatives and dairy consumed, other research suggests that populations with high relative disadvantage demonstrate low diet quality scores for dairy, notably cheese, yogurt and dairy alternatives and lean meat and meat alternatives^(39)^. These differences in findings between studies could potentially stem from the under-representation of rural and regional participants in other studies. However, an alternative explanation is that rural populations may prefer fatty cuts of meat and dairy spreads^(51)^, possibly influenced by a greater availability of these items in rural and regional areas^(23)^. Other research has identified meat and dairy as having higher priority in the budget of low-income households due to perceived value as the primary foundation of a complete meal^(44)^.

Strengths of the current study include an adequately powered sample of rural and regional Australian adults, and a validated tool was used to measure diet quality, which measures variety from, within and between the food groups^(52)^. However, the ARFS has potential limitations, such as not accounting for discretionary food intake or distinguishing between refined grains and wholegrains, as well as lean versus high fat and processed meat options. These dietary factors, often linked to area-level disadvantage^(41)^, may limit the tool’s ability to fully capture the complexity of associations between socio-economic position and diet quality in this study. A further limitation could be using postcode to define socio-economic position rather than suburbs. The research has shown that the larger the geographical area represented by an index of disadvantage, the greater the potential for misclassification^(53)^. With respect to the methodologies applied in the study, it can be anticipated that the utilization of non-probability sampling may introduce a certain level of sampling bias. This sampling bias may have influenced our findings, potentially leading to an overrepresentation of individuals with higher socio-economic status in our sample. As a result, the relative distribution of social advantage and disadvantage in our sample may differ from that of the broader Tasmanian population. Further, not making the dietary section mandatory may have introduced further socio-demographic bias, for example those with higher education may have been more inclined to continue to complete the voluntary diet section of the questionnaire. The cross-sectional design of the present study prevents us from determining causal relationships between socio-economic position, characterised by area-level relative advantage and disadvantage and diet quality. Further, the relatively small geographical area captured in our sample means that our results potentially lack generalisability to the broader population.

### Conclusion

The current results demonstrate that residing in areas with low socio-economic position is associated with lower diet quality in rural and regional Australian adults. The results demonstrated that while there was no significant difference in diet quality scores between quintiles 2–5 of SEIFA IRSAD, quintile 1 participants had significantly lower diet quality scores. Further, we found that low diet quality in this subgroup appears to be driven by low ARFS subscale scores for variety of fruit, vegetables and grains. This study also found that these differences remained significant when controlling for individual-level socio-economic variables. Considering this, further research investigating differences in the relative access to social and economic resources specific to those living in geographical locations that are characterised by low socio-economic position is warranted. Examination of the sources of disparity between population groups defined as being of low socio-economic position, specifically those in the most disadvantaged 20 % of regions, may assist in developing focused strategies to increase consumption and variety of fruit, vegetables and grains in order to improve overall diet quality in this population. Given that scores for most food groups in the present sample were poor, such efforts may benefit from taking a comprehensive approach to improving diet rather than focusing solely on individual aspects of diet. Future research should further characterise dietary intake by investigating dietary consumption which extends beyond the five food groups of the Australian Dietary Guidelines, with suggested focus on dietary patterns and inclusion of discretionary food intake, including intake of ultra processed foods. Further, future investigation should also consider adopting recruitment methods that disaggregate populations into smaller measurable units, such as suburbs. In addition, future research exploring the specific barriers and enablers faced by populations of low socio-economic position, such as the local food environment and the impact of built environment and infrastructure on diet quality, particularly in rural and regional areas is needed. The present study has found an independent effect of area-level socio-economic position on diet quality. However, there were significant differences in education and income in our sample that warrant further investigation to tailor specific and meaningful interventions designed to increase diet quality in those population groups facing high relative disadvantage.

## Supporting information

Pullen et al. supplementary material Pullen et al. supplementary material

## Supplementary material

For supplementary material/s referred to in this article, please visit https://doi.org/10.1017/S0007114525103486
