# Supplementary material for: The association between socio-economic position and diet quality in rural and regional Australian adults
Source: Br J Nutr. 2025 Jun 30;133(12):1532–42. doi: 10.1017/S0007114525103486 (PMC12335914; doi:10.1017/S0007114525103486)
Supplement: Pullen et al. supplementary material [file S0007114525103486sup001.docx]

**Table 1**. **Pairwise comparison of mean total Australian recommended Food Score (ARFS) for SocioEconomic Indexes for Areas (SEIFA), Index of Relative Social Advantage and Disadvantage (IRSAD) quintiles.**

| **SEIFA IRSAD Quintile** | **Comparison SEIFA IRSAD Quintile** | **Mean difference** | **SE** | **95% CI** | **p-value** |
| --- | --- | --- | --- | --- | --- |
| 1 | 2 | -4.1* | 1.1 | [-7.3, -0.9] | 0.003 |
|  | 3 | -4.9* | 1.2 | [-8.2, -1.5] | <0.001 |
|  | 4 | -5.7* | 1.1 | [-8.8, -2.7] | <0.001 |
|  | 5 | -5.2* | 1.0 | [-8.0, -2.4] | <0.001 |
| 2 | 1 | 4.1* | 1.1 | [0.9, 7.3] | 0.003 |
|  | 3 | -0.8 | 1.1 | [-3.9, 2.4] | 1.0 |
|  | 4 | -1.6 | 1.0 | [-4.5, 1.3] | 1.0 |
|  | 5 | -1.1 | 0.9 | [-3.6, 1.5] | 1.0 |
| 3 | 1 | 4.9* | 1.2 | [1.5, 8.2] | <0.001 |
|  | 2 | 0.8 | 1.1 | [-2.4, 3.9] | 1.0 |
|  | 4 | -0.9 | 1.1 | [-3.9, 2.2] | 1.0 |
|  | 5 | -0.3 | 1.0 | [-3.1, 2.2] | 1.0 |
| 4 | 1 | 5.7* | 1.1 | [2.7, 8.8] | <0.001 |
|  | 2 | 1.6 | 1.0 | [-1.3, 4.5] | 1.0 |
|  | 3 | 0.9 | 1.1 | [-2.2, 3.9] | 1.0 |
|  | 5 | 0.5 | 0.9 | [-1.9, 3.0] | 1.0 |
| 5 | 1 | 5.2* | 1.0 | [2.4, 8.0] | <0.001 |
|  | 2 | 1.1 | 0.9 | [-1.5, 3.6] | 1.0 |
|  | 3 | 0.3 | 1.0 | [-2.5, 3.1] | 1.0 |
|  | 4 | -0.5 | 0.9 | [-3.0, 1.9] | 1.0 |

* Significance of mean difference set at 0.05
